# SARS-CoV-2: Overview and Its Impact on Oral Health

**DOI:** 10.3390/biomedicines9111690

**Published:** 2021-11-15

**Authors:** Miriam Ting, Jon B. Suzuki

**Affiliations:** 1 Think Dental Learning Institute, Paoli, PA 19301, USA; 2 School of Medicine and School of Dentistry, University of Maryland, Baltimore, MD 20742, USA; jon.suzuki@temple.edu

**Keywords:** ACE2, COVID-19, oral hygiene, periodontitis, bacteria, SARS-CoV-2, periodontal, coronavirus

## Abstract

The severe acute respiratory syndrome coronavirus 2 (SARS-CoV-2) and its virulent variants causing coronavirus disease 2019 (COVID-19) COVID-19 has spread rapidly worldwide, and is highly contagious. A comprehensive search was conducted for the most current published information about SARS-CoV-2, COVID-19, and oral health. Clinical studies, case reports, in vivo studies, and any current published evidence on SARS-CoV-2 and COVID-19 were included in this review. Survival against SARS-CoV-2 infection may be partially dependent on periodontal health, good oral hygiene, and access to dental care. Optimum oral health, maintaining good systemic health, and elimination of smoking habits may be beneficial for the prevention and management of COVID-19 infections.

## 1. Introduction

The severe acute respiratory syndrome coronavirus 2 (SARS-CoV-2) outbreak in Wuhan, China, was first reported to the China Ministry of Health and the World Health Organization (WHO), on 31 December 2019. These initial cases were linked to an animal wet market. The SARS-CoV-2 and its virulent variants cause coronavirus disease 2019 (COVID-19), which is highly contagious and spreads rapidly. The WHO declared it a global emergency on 30 January 2020, and a global pandemic on 11 March 2020. The SARS-CoV-2 genome was identified to be 70–80% identical to the severe acute respiratory syndrome coronavirus (SARS-CoV) and several bat coronaviruses [1]. The similarity between these coronaviruses suggests that the bat may be the natural host and potential reservoir for SARS-CoV-2, which may have been inadvertently transmitted to humans [2]. SARS-CoV-2 transmits readily via droplet transmission; other modes of transmission may include aerosol and oral-fecal routes [3]. It can also be transmitted via contact with infected surfaces and oral fluids [4]. This puts dentists and healthcare professionals at risk of COVID-19 infections, and highlights dental and medical offices as a main risk setting for cross infection of patients and healthcare professionals [5]. Mask, face shields, handwashing, and personal protective equipment (PPE) are currently used in dental and medical facilities for COVID-19 prevention consistent with CDC guidelines.

## 2. Pathogenesis and Cellular Mechanism of Action

**Figure 1:**
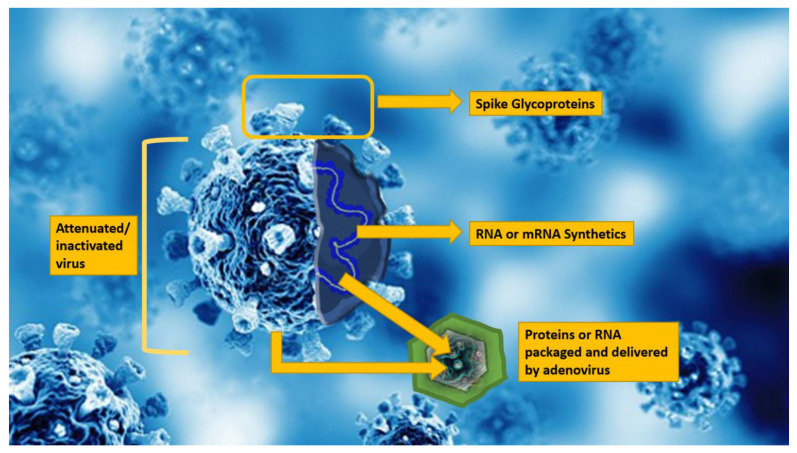
Artist sketch of SARS-CoV-2 virus depicting spike protein and mRNA core.

SARS-CoV-2 comprises a single stranded RNA with cell-surface spike glycoproteins, which facilitate adherence and penetration of host cells (Figure 1) [6]. The main cellular receptor for the SARS-CoV-2 spike glycoprotein is the angiotensin-converting enzyme 2, found in the lungs, kidneys, myocardial cells, salivary glands, and tongue.

**Figure 2:**
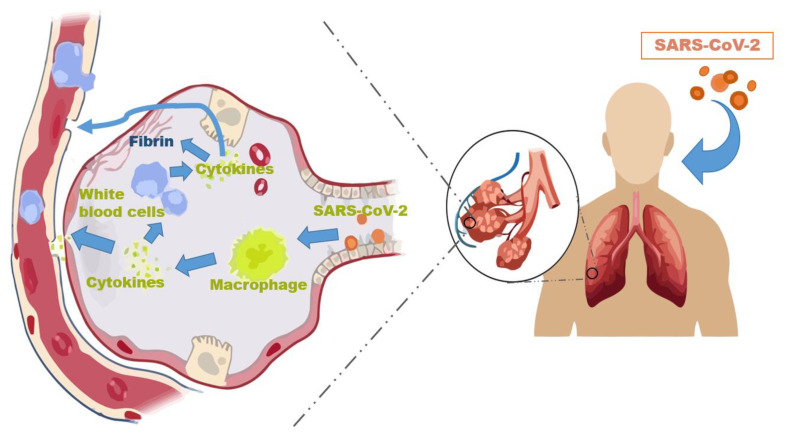
Proposed pathogenesis after SARS-CoV-2 enters the pulmonary tree. SARS-CoV-2 is engulfed by local alveolar immune cells and triggers an immune response cascade. Local immune cells are activated and release varying quantities and types of cytokines. The released cytokines, especially during cytokine storms, have both a local and systemic effect on the host.

The clinical presentation of SARS-CoV-2 infections are often asymptomatic or have mild to moderate symptoms; 5% or less develop multi-organ failure or acute respiratory distress syndrome. An epidemiologic study reported 17% of COVID-19 patients are asymptomatic, and that asymptomatic patient transmission of COVID-19 is statistically similar to the symptomatic patient [7]. The disease can progress in 3 main stages. Stage 1 involves the activation of innate immunity. Stage 2 involves the activation of adaptive immunity. Stage 3 involves the cytokine release syndrome or the “cytokine storm” [8]. The cytokine storm (Figure 2) is an exaggerated cytokine release by a hyper-responsive host [9,10]. It is characterized by hyper-coagulability, dysfunction of multiple organs, acute lung injury, and shock [8]. The enhanced vascular permeability facilitates effector cells infiltration, and intensifies pro-inflammatory cytokine release. This cytokine release can induce excessive monocyte proliferation and lymphocyte apoptosis, resulting in potential immunodeficiency states [11].

Pro-inflammatory cytokines such as IFN-γ, IFN-γ-induced protein 10, IL-1, IL-6, IL-12, and monocyte chemoattractant protein were reported in earlier studies of SARS-CoV-2 infected patients [12]. Recent studies reported that elevated serum levels of IL-6 were positively associated with disease severity [13,14,15,16,17] and mortality in older patients and patients with comorbidities [18]. Dysregulation of immune functions and overproduction of early response pro-inflammatory cytokines can lead to multi-organ failure, especially of the heart and kidneys [19,20,21]. The IL-6 levels in non-survivors are higher than in SARS-CoV-2 survivors [10].

**Figure 3:**
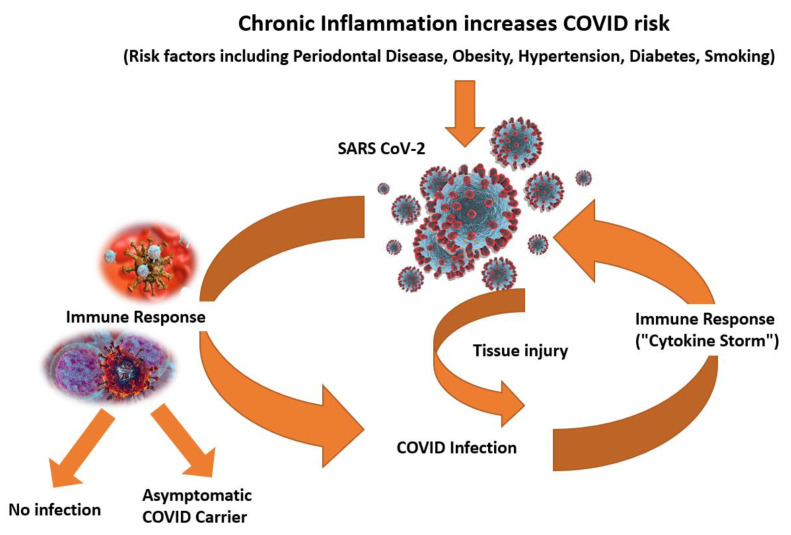
Chronic inflammatory diseases and conditions such as uncontrolled hypertension, diabetes, obesity, smoking, and periodontal diseases, among others, appear to significantly increase risk for COVID-19 infections and may influence pathogenesis.

Periodontal inflammation, among other chronic inflammatory diseases and conditions, may influence COVID-19 susceptibility and pathogenesis (Figure 3). Three different clinical responses in the periodontium to bacterial burden may shed light on COVID clinical responses to SARS-CoV-2 virus challenges. These host inflammatory clinical responses to oral bacteria were designated as “high”, “low”, and “slow” [22]. The high clinical response group to similar bacterial concentrations resulted in high IL-1β levels in inflamed tissues. Applying this model to COVID-19 infected individuals, the varying levels of inflammatory response may be a plausible explanation for non-vaccinated individuals being at varying levels of risk for pulmonary “cytokine storms”, resulting in outcomes from hospitalizations to possible death [22].

## 3. Systemic Ramifications

Clinical symptoms of SARS-CoV-2 appear approximately 5.2 days following infection [23]. Symptoms reported were fever, fatigue, myalgia, diarrhea, dry cough, sore throat [24,25], and loss of taste [26]. During the initial days after SARS-CoV-2 infection, COVID patients primarily appear asymptomatic; the virus colonizes the oral, nasal, and pharyngeal mucosa, and is highly infectious [27].

Risk factors of SARS-CoV-2 were highlighted by Zhou et al. in a retrospective cohort study of COVID-19 patients [28]. Some of the risk factors cited were advanced age, male gender, hypertension, diabetes, heart disease, and obesity [1,28,29]. Complications of severe SARS-CoV-2 infection were blood clots, sepsis, septic shock, pneumonia, and acute respiratory distress syndrome (ARDS) [30]. In severely infected patients in China, 41.8% developed ARDS. Of these patients with ARDS, respiratory failure resulted in 52.4% mortality [31]. In infected patients in Italy, 96.5% of reported complications were ARDS, followed by 29.2% acute renal failure [32]. Thus, these post-viral complications, especially ARDS, were likely the cause of death rather than the initial viral infection. Most respiratory viral infections increase the patient’s vulnerability to respiratory bacterial superinfections and inflammatory lung damage [33]. 

Antibody response to SARS-CoV-2 viral antigen, nucleoprotein, and spike protein peaks 14–21 days after symptom onset [34]. In the oral cavity, immunoglobulin A (IgA) dominates early mucosal immune response [35]. Individuals who have who have been exposed to the SARS-CoV-2 virus appear to have high levels of neutralizing secretory antibodies.

## 4. Long COVID

The median time from onset of COVID-19 to recovery is approximately 2 weeks for mild infections, and 3–6 weeks for severe disease [36]. Long COVID can develop when SARS-CoV-2 symptoms persist beyond 4 weeks after COVID-19 diagnosis. A retrospective cohort study reported that 34% of patients had persistent psychiatric or neurological symptoms six months following COVID-19 disease onset [37]. Another study reported 87.4% of hospitalized COVID-19 infected patients have symptoms 60 days after initial diagnosis [38]. Long COVID or post-COVID-19 syndrome differs from acute COVID-19, in that it has a higher prevalence in women and was highest in patients aged 24–36 years old. Long COVID symptoms include fatigue, brain fog, fever, coughing, breathlessness, and muscle and joint pain [39]. 

Many of COVID-19-related systemic conditions may persist during long COVID [40]. In an examination of the recognized systemic extensions of SARS-CoV-2, multiple organs and their functions are implicated in primary COVID infections [41]:

The recognized neurological clinical signs and symptoms impacting on the brain include memory loss, confusion and difficulty focusing (“brain fog”). More severe symptoms include depression, psychosis, and anxiety. 

Taste and smell are frequently reported to be impaired. Taste and smell sensations are common in primary COVID-19 infections, but not consistently reported in long COVID. However, the potential of headaches, encephalitis, and strokes require constant monitoring of long COVID patients.

Myocarditis, and the inflammation of cardiac muscle, have been reported in primary COVID-19 infections. Other cardiac signs and symptoms include arrhythmias, heart damage, and failure. Patients with long COVID-19 reported having dyspnea, angina, and palpitations.

Metabolic panel surveys of blood samples taken from primary COVID-19 patients exhibit abnormal liver enzymes. Whether these have a clinical impact on coagulation has not been determined. The liver metabolic panel surveys in long COVID-19 patients have also not been determined to date. Pre-existing liver conditions such as cirrhosis, Hepatitis B, and fibrosis appear to serve as a comorbidity and an elevated risk of liver dysfunction.

Long COVID patients consistently report coughing, dyspnea, and fatigue. These signs and symptoms continue into long COVID disease and, are perhaps related to persistent cytokine production by inflammatory cells in the lungs. Cytokine storms certainly accelerate signs and symptoms, and may result in death. However, long COVID is most likely due to continued cytokine levels being elevated, e.g., IL 1-β, TNF-α, IL-6, among others. The microthrombi to the larger blood clots in the vessels of selected organs, including the lungs, appear to be the key mechanism underlying the broad sweeping systemic effects on the body by COVID-19.

Loss of appetite, diarrhea, bowel blockages, and nausea are frequent systemic manifestations of COVID-19 in the gastrointestinal tract. The data are inconsistent regarding the extension of these signs and symptoms in long COVID.

There are elevated glucose levels, hyperglycemia, among diabetic mellitus patients with COVID-19, implicating the pancreas. The data are inconsistent regarding the extension of observed serum glucose levels in long COVID.

SARS-CoV-2 infection can cause long-standing damage to the immune system characterized by increased inflammatory cytokine activation [42]. Vaccines provide significant reduction in breakthrough infections with COVID-19. United Kingdom data reported [43] that fully vaccinated patients are at a reduced risk for long-haul COVID, serious complications, and breakthrough infections. Patients that received the first dose of Pfizer-BioNTech, Moderna, or AstraZeneca–Oxford vaccines have 0.5% risk of a breakthrough infections after 14 days.

After the second dose of these vaccines, 0.2% of subjects report a COVID breakthrough infection. The UK report further supports patients receiving both doses of the two-dose vaccine regimen, with 94% of asymptomatic patients after receiving both doses.

## 5. Immunosenescence

The highest morbidity and mortality from COVID-19 was reported in adults aged 70 years and older [44]. Children appear to be less affected by COVID-19 [45]. Age-related effects on the immunity (immunosenescence) may explain increased mortality of SARS-CoV-2 in older populations. Immunosenescence of both innate and adaptive immunity may result in increased cytokine secretion [46], impairment of lymphocyte blastogenic responses [47], ineffective T-cell response, failed antibody production, and inflammation related severe organ dysfunction [48]. Thus, immunosenescence may increase susceptibility to infectious disease, increase the severity of disease, and reduce responses to vaccine.

Recently, the CDC reported that vaccine effectiveness wanes with selected medical conditions. Immunocompromised patients and patients older than 65 years exhibit decreased susceptibility to COVID-19. In this study, although highly effective at 24 weeks, it appears that immunosenescence for the two vaccines (Moderna and Pfizer-BioNtech) may result in higher risks of COVID-19 breakthrough infections in these patient groups [49].

COVID-19 vaccine break through infections were reported August 6, 2021 by the CDC [50]. These breakthrough infections could be due to waning of the vaccine antibody response or the emergence of variants of SARS-CoV-2. The CDC (MMWR 6 August 2021) reported 469 cases of COVID-19 among individuals in a Massachusetts town, who participated in a relatively large gathering. The Delta variant was identified in 90% of the patient specimens from 133 patients. Fully vaccinated patients had similar antibody titer, compared to non-vaccinated patients.

Patients with a positive COVID test result have been reported to have prevalence of long-term symptoms of SARS-CoV-2 infection [50]. Patients testing negative have symptoms persisting for less than 4 weeks. Non-hospitalized positive SARS-CoV-2 infected patients have a higher prevalence of long-term symptoms or conditions than non-hospitalized patients with negative SARS-CoV-2 test results [51].

## 6. Vaccines

The ideal SARS-CoV-2 vaccine should be effective after 1-2 vaccinations, be able to protect vulnerable populations (elderly, immunocompromised, or other co-morbidities), protect for at least six months, and reduce viral transmission. The minimum criterion of at least 50% vaccine efficacy is required to be considered a successful vaccine by the US Food and Drug Administration (FDA) guidance document [52]. Vaccine efficacy in randomized controlled trials evaluated the following parameters: infection reduction, reduction in severity of clinical disease, and reduction of infectivity duration [53]. The protection attributed to the vaccine is reported as a proportional reduction in infection between subjects that were vaccinated and a control group. However, efficacy does not always equate effectiveness. Other factors such socioeconomic conditions, particular age groups, geographical settings, and herd immunity may interfere with outcome data.

For SARS-CoV-2, an effective vaccine may prevent infection, disease progression, or disease transmission. The desired outcome of an effective SARS-CoV-2 vaccine is to protect against death and disease severity. Most SARS-CoV-2 vaccines aim to neutralize either the spike protein, mRNA, or attenuated/inactivated virus [54]. Clinical trials for COVID-19 vaccines investigate the neutralizing antibody response; types of vaccine evaluated include mRNA [55,56], adenoviral vector [57,58], spike glycoprotein [59] with adjuvants, and inactivated virus [60,61]. 

Although there are multiple approaches for vaccine development [62] to SARS-CoV-2, four primary methodologies have emerged: nucleic acid DNA or m-RNA, spike proteins (protein subunits), adenovirus carrier (viral vector), and inactivated (whole) virus. The current vaccines available worldwide for m-RNA vaccines included Pfizer-BioNTech and Moderna; for viral-vector vaccines included Oxford–AstraZeneca, Johnson and Johnson—Janssen and Sputnik V, and inactivated virus vaccines include Sinovac (Table 1). 

Other SARS-CoV-2 vaccines continue to be developed aggressively. As of the date of publication, 121 COVID-19 vaccine candidates have clinical trials in phase 3. In other laboratories, 194 candidates, are in pre-clinical development around the world. For example, SARS-CoV-2 vaccines are in development by a Canadian company (Medicago) and an Indian company (Biological E); both companies have proceeded to phase 3 trials. Another pharmaceutical company, TURKOVAC, developed by Turkish Kocak Farma and Health Institutes of Turkey, has registered phase 2 trials.

Viral-vectored vaccines are highly immunogenic, potent inducers of antibodies and cytotoxic T-cells [63,64], and have the added advantage of inducing strong responses in older adults and immunocompromised individuals [65,66]. The SARS-CoV-2 spike RNA vaccine is a potent stimulator of T-helper cells (CD4) and T-cytolytic cells (CD8), which can improve protection against severe COVID-19 [56,67].

The BNT162b2 mRNA vaccine (Pfizer) may provide early protection 12 days after first dose [68,69], and is approximately 95% efficient against COVID-19 seven days after the second dose. The viral load is significantly reduced 12 days after vaccination, and may limit the disease severity and transmission of COVID-19 [70].

The large-scale phase 3 clinical trials of BNT162b2 and mRNA-1273 vaccines showed a high vaccine efficacy of 94–95% for the duration of several months, and can attest to short-term vaccine efficacy [68]. 

Vaccine efficacy can decrease over time due to mutating antigenicity or diminishing immunologic memory. It is important to consider vaccine boosters to increase the longevity of protection. The use of two vaccinations or multiple vaccine types may provide robust and longer lasting immunity [71,72]. 

Individuals who have been immunized with SARS-CoV-2 vaccine appear to have high levels of neutralizing secretory IgA antibodies. According to this study, individuals who are immunized may not be able to transmit SARS-CoV-2 [73]. On the contrary, new preliminary evidence suggests that immunized patients maybe be able to carry or transmit SARS-CoV-2 [74]. 

## 7. Drug and Intravenous Therapy

For plasma therapy, clinical trials evaluated the use of SARS-CoV-2 convalescent plasma from recovered COVID-19 patients [75]. The SARS-CoV-2 convalescent plasma contains antibodies that can potentially treat life-threatening COVID-19 infected patients. In a case series of five critically ill patients who were administered SARS-CoV-2 convalescent plasma, the symptoms diminished in 10 days [76]. However, these case reports are limited in evidence, and would require further randomized clinical trials. COVID-19 convalescent plasma is available for hospitalized patients through the FDA Emergency Use Authorization (EUA).

Recent randomized trials of convalescent plasma therapies have been suspended before clinical endpoints were completed. Although clinical randomized trials have not been completed to date, convalescent plasma appears to be of benefit for selected patients with COVID-19 [77].The potential benefit to critically ill patients has been reported to be 2.2% [78]. In principle, donor neutralizing antibodies present in convalescent plasma inactivates SARS-CoV-2 virus in the recipient [79]. Potential biologic variation in donor convalescent plasma may account for discrepancy in the clinical outcomes for these COVID-19 patient.

For neutralizing monoclonal antibodies, a bamlanivimab and etesevimab combination therapy may reduce viral load and emergent resistant variants [80]. In patients with mild to moderate COVID-19, bamlanivimab and etesevimab combination therapy significantly reduced SARS-CoV-2 viral load at day 11, compared to the control group [81]. Furthermore, the FDA expanded the EUA to allow bamlanivimab and etesevimab combination, and casirivimab plus imdevimab combination, and sotrovimab to be used as post-exposure prophylaxis for patients who were at high risk for progression of severe COVID-19 [82].

For drug therapy, a “solidarity” clinical trial [83] for COVID-19 treatment reported by the WHO evaluated a nucleotide analogue Remdesivir, a malaria medication chloroquine and hydrochloroquine, and a combination of anti-HIV medications (lopinavir and ritonavir) with or without interferon-b.

Remdesivir can inhibit emerging coronavirus RNA chains, by inhibiting RNA-dependent RNA polymerases and competing for the adenosine needed for viral incorporation [84]. It is FDA approved for treating severely ill COVID-19 patients.

Chloroquine or hydrochloroquine can inhibit viral enzymes (RNA polymerase), various steps of viral replication, and ACE-2 receptors. It can also decrease acidity in endosomes and immunomodulate cytokine release [85]. 

A combination of anti-HIV medications (lopinavir-ritonavir) can inhibit M protease, an essential enzyme needed for coronavirus replication [86]. However, other reports cautioned its use as treatment outcomes were not significantly different compared to a control group [87]. 

A combination of lopinavir-ritonavir and interferon-b can further activate the innate antiviral response. However, this may also further exacerbate inflammation causing detrimental effects [88]. 

On the other hand, Molnupiravir is the first direct-acting antiviral with an oral route of administration. Molnupiravir acts by disrupting RNA viral duplication. Oral administration of molnupiravir may be a significant advantage over current intravenous COVID-19 therapies such as Remdesivir, which require intravenous infusion for 3 days. Phase 2a trial preprint data [89] of molnupiravir treatment reported no SARS-CoV-2 detected in patients receiving 400 or 800 mg molnupiravir, compared to 11.1% of those receiving a placebo. Adverse reactions to Molnupiravir were reported to be far less than COVID-19 signs and symptoms. No evidence of mutagenesis in either cell lines or animal models were observed. The delivery of anti-viral oral medications such as Molnupiravir for COVID-19 treatment may reduce hospitalizations, and may be a significant advantage over currently available IV therapies. 

## 8. Targets for SARS-CoV-2 Entry in the Oral Cavity

SARS-CoV-2 infectivity depends on its ability to penetrate the cell. SARS-CoV-2 uses the angiotensin-converting enzymes 2 (ACE2) receptor for cellular entry [90]. The ACE2 receptors were present in oral mucosa tissues including the floor of the mouth, tongue, buccal mucosa, and gingiva [91]. The oral ACE2-positive cells reside mainly in the taste organs [91]. Loss of taste has been commonly reported [26,92], with 91% of patients experiencing this before hospitalization due to COVID-19 [91]. 

In the early stage of COVID-19 infection, SARS-CoV-2 has been consistently detected in whole saliva [93]. ACE2-positive salivary glands are also targets for SARS-CoV-2, and may affect salivary gland function [94]. In Wuhan, 46% of COVID-19 infected patients reported a dry mouth [26]. The ACE2 receptors in the salivary glands is higher than the lungs, and is a suggested reservoir for SARS-CoV-2 in asymptomatic patients [95]. 

The ACE2 receptors are also present in fibroblasts in the periodontium [96], and elevated protease levels due to chronic periodontitis can increase risk for viral entry [97]. While the pulmonary system remains the primary modality for infectivity by SAR-CoV-2, it is plausible that selected components of the oral cavity may be a contributing factor.

In addition, the S protein of the SARS-CoV-2 needs to be cleaved by transmembrane protease serine 2 (TMPRSS2) or furin to enable fusion to the host cell [98,99,100]. Besides TMPRSS2, furin, or ACE2 in the oral cavity [28,101], pathogenic bacteria found in the oral cavity can also cleave the S protein of the SARS-CoV-2 [102]. 

## 9. Effects of Oral Health on COVID-19

Oral health affects overall health and well-being [103]. Since the oral cavity is one of the interfaces that connects to the exterior of the body, the ability of SARS-CoV-2 to utilize this interface for entry will determine its infectivity [91,95]. The health of the oral cavity and its structures may contribute to increased or decreased risk of COVID-19 [91,95]. A healthy oral cavity consists of a symbiotic balance of gram-positive bacteria. Poor oral hygiene and periodontitis can tip this balance towards dysbiotic biofilms that promote cytokine release. This elevated level of cytokines may have proinflammatory systemic effects, and may have a role in propagating pulmonary infections [104]. In addition, the interbacterial exchange of pathogenic bacteria from the oral cavity to the lung may contribute directly to lung infections [105]. Poor oral hygiene and the aspiration of periodontal pathogens can aggravate COVID-19 [102]. Aspirated bacteria may cause inflammation of the lower respiratory tract and exacerbate COVID-19. In patients with severe COVID-19, half were reported to die of secondary bacterial infections rather than from the virus [28]. This bacterial superinfection can supersede the original COVID-19 infection. Patients with severe COVID-19 present with higher neutrophils compared to lymphocytes. Higher neutrophil counts have been indicative of bacterial infections rather than viral infections [106]. 

In the medically compromised and the elderly, the increased risk of bacterial aspiration due to a poor swallowing reflex [107] may increase the severity of COVID-19 [108,109]. Periodontal bacteria are not indigenous of the lower respiratory bacterial flora, but have been isolated in patients with COVID-19 [110]. Poor oral hygiene increases periodontal pathogens, which can raise expression of ACE2, increase pro-inflammatory cytokines, and degrade the S-protein. *F. nucleatum* can upregulate ACE2 transcription, and induce IL-8 and IL-6 production in alveolar epithelial cells [111]. The degradation of the S protein by microbial proteases may increase SARS-CoV-2 penetration and infectivity [99,100]. Moreover, the lack of proper oral care in COVID-19 patients on long-term hospitalization may increase the risk of aspirated pathogenic oral bacteria and inflammation in the lower respiratory tract. Thus, the increased prevalence of pathogenic bacteria associated with poor oral hygiene may contribute to the progression of COVID-19 via upregulation of ACE2 and proinflammatory cytokines [102]. 

Chronic inflammation from periodontitis may also increase the risk of more severe COVID-19 outcomes. In a survey from 2009–2014 of adults older than 30 years, 42% had periodontitis [112]. Periodontal disease and associated co-morbidities, including chronic obstructive pulmonary disease, diabetes mellitus, hypertension, and cardiovascular and cerebrovascular disease, can worsen the COVID-19 prognosis [113]. According to the CDC, diabetes and cardiovascular disease are the most prevalent underlying comorbidities among those hospitalized due to COVID-19 [114]. 

COVID-19 patients with periodontal disease have a higher mortality risk than patients without periodontitis [115]. The immune cellular release of cytokines including IL-1 and TNF in periodontitis may contribute and exacerbate the recognized “cytokine storms “associated with COVID-19 infections.

Thus, oral hygiene plays a significant role; non-optimal brushing may result in increased levels of gingival inflammation and higher cytokine levels. Higher cytokine levels may increase COVID risk. It is reasonable to assume that the management and control of periodontitis-induced destructive cytokines may reduce or minimize the risk of SAR-CoV-2 infections.

## 10. Current Preventive Strategies in the Oral Cavity

Oral antiseptics used as pre-procedural rinses, to reduce the risk of cross-infection and the amount of bacteria in aerosols, have been shown to be effective. A meta-analysis evaluating the effectiveness of pre-procedural mouth rinses reported a reduction in the number of aerosolized microbes during dental treatment [116]. Other studies evaluated oral antiseptics indirectly by reporting on in vitro antiviral activity [117]. Oral antiseptics can reduce viral load and disease transmission, by disrupting the viral lipid envelope [117]. Selected oral antiseptics used to prevent viral cross-contamination include 1% povidone-iodine [118], 0.05–0.10% cetylpyridinium chloride [119], 0.12% chlorhexidine [120], 1% hydrogen peroxide [121], beta-cyclodextrin with citrox [122], and essential oil mouth rinses (e.g., eucalyptol, thymol, menthol, methyl salicylate) [123]. A combination of two mouth rinses, 1% hydrogen peroxide, and 0.2–0.3% chlorhexidine can also be advantageous in utilizing two active ingredients in sequence for dual mechanism of action [124]. These antiseptic rinses can decrease salivary viral load and reduce the risk of SARS-CoV-2 dissemination [125]. 

A recent randomized control clinical trial explored the efficacy of antimicrobial mouth rinses when rinsed for 60 s in reducing viral load in asymptomatic SAR-CoV-2 patients [126]. It is likely that pre-symptomatic and post-asymptomatic patients form a minor but significant portion of patients seeking dental therapies. This randomized triple blinded study evaluated chlorhexidine (0.12%), povidone iodine (0.5%), and hydrogen peroxide (1%), with sterile saline as a control. The PCR viral load was measured 15- and 45-min post-rinsing. All four mouth rinses, including the saline control, decreased viral load from 61% to 89% at 15 min post-rinsing, and 70% to 97% at 45 min. SARS-CoV-2 viral copies were measured using real time reverse transcriptase quantitative PCR. According to this study, antimicrobial rinses may be a productive means to reduce salivary viral risk in a dental practice utilizing antimicrobial pre-rinsing. 

The major limitation of this study is that primarily SARS-CoV-2 viral loads were evaluated, in exclusion of other respiratory viruses and oral bacteria. A dental practice must also take into consideration preventing the overwhelming bacterial load of the oral cavity in aerosol sprays emanating from dental and hygiene procedures. This published study may suggest that povidone iodine, hydrogen peroxide, and chlorohexidine may be the optimal antiviral and antibacterial rinse. However, another study also suggests that cetypyridinium chloride, povidone iodine, and chlorohexidine exhibits optimal anti-SARS-CoV-2 activity [121]. Perhaps substantivity and long-term bioactivity of antimicrobial mouth rinses must also be taken into consideration for the dental practice.

Thus, gargling antimicrobial mouthwashes or the use of antimicrobial nasal sprays in suspected or confirmed COVID-19 patients may inhibit transmission of infection and protect healthcare providers [127]; however, more completed data from these ongoing studies are required.

Optimal oral hygiene and treatment of periodontal disease can reduce ACE2 expression, inflammatory cytokines, and aspiration pneumonia [128]. Thus, maintaining periodontal health may reduce host susceptibility to COVID-19, and may prevent COVID-19 aggravation in infected patients [102]. Periodontal disease therapy also improves systemic diseases such as COPD and diabetes [129,130]. Ideal dental health may reduce mortality and morbidity due to pneumonia and influenza, respectively [131,132]. Meticulous oral hygiene may reduce ACE2 expression and decreased inflammatory cytokine release. Thus, preventing aspiration pneumonia and COPD by the management of oral hygiene may lower host susceptibility to COVID-19. In addition, for SARS-CoV-2 infected patients, maintenance of good oral conditions may lead to prevention of COVID-19 aggravation. Thus, periodontal disease therapy and maintaining good oral hygiene are crucial for overall health.

## 11. Conclusions

Overall health and survival against SARS-CoV-2 may partly be dependent on periodontal health and good oral hygiene. In addition to maintaining good systemic health and the elimination of smoking habits, optimum oral health may be beneficial for prevention and management of COVID-19 infections. It may also be speculated that the use of antimicrobial mouth rinses may become routine therapeutic agents used to enhance oral health, and inhibit the transmission of COVID-19 in the dental office.

## Figures and Tables

**Table 1:** Major vaccines for SAR-CoV-2 available worldwide (updated November 2021).

Vaccine	Type	Doses	Booster	Age Group
**Pfizer-BioNTech**	m-RNA	2 (30 ug/mL, 3 wks apart) (>12 yrs) (10 ug/mL, 3 wks apart) (5–11 yrs)	Yes	Adults (>18 yrs) Teens (12–18 yrs) Children (5–11 yrs)
**Moderna**	m-RNA	2 (100 ug/mL, 4 wks apart) (>18 yrs) TBD (6–18 yrs)	Yes	Adults (>18 yrs) Teens (12–18 yrs) Children (6–11 yrs)
**J & J-Janssen**	Viral vector	1 (0.5 mL)	Yes	Adults (>18 yrs)
**Oxford–AstraZeneca**	Viral vector	2 (0.5 mL, 8–12 wks apart)	TBD	Adults (>18 yrs)
**Sputnik V**	Viral vector	2 (0.5 mL, 3 wks apart)	Yes	Adults (>18 yrs)
**Sinovac**	Inactivated virus	2 (0.5 mL, 2–4 wks apart)	Yes	Adults (>18 yrs)
